# Effect of Antimicrobial Filler on Ethylene-Vinyl Acetate (EVA) Composites Property

**DOI:** 10.3390/ma18214993

**Published:** 2025-10-31

**Authors:** Kamil Kwieciński, Marta Chrószcz-Porębska, Izabela Barszczewska-Rybarek, Jarosław Żmudzki, Anna Mertas, Sebastian Jurczyk, Paweł Popielski, Grzegorz Chladek

**Affiliations:** 1 Apipharma Sp. z o. o., 44/7 Kościuszki Str., 40-040 Katowice, Poland; 2 Chair of Drug and Cosmetics Biotechnology, Faculty of Chemistry, Warsaw University of Technology, 3 Noakowskiego Str., 00-664 Warsaw, Poland; marta.porebska@pw.edu.pl; 3 Department of Physical Chemistry and Technology of Polymers, Faculty of Chemistry, Silesian University of Technology, Strzody 9 Str., 44-100 Gliwice, Poland; izabela.barszczewska-rybarek@polsl.pl; 4 Department of Engineering Materials and Biomaterials, Faculty of Mechanical Engineering, Silesian University of Technology, 18a Konarskiego Str., 41-100 Gliwice, Poland; jaroslaw.zmudzki@polsl.pl; 5 Department of Microbiology and Immunology, Faculty of Medical Sciences in Zabrze, Medical University of Silesia in Katowice, 19 Jordana Str., 41-808 Zabrze, Poland; amertas@sum.edu.pl; 6 Łukasiewicz Research Network—Institute of Polymer Materials, 55 Skłodowskiej-Curie Str., 87-100 Torun, Poland; sebastian.jurczyk@impib.lukasiewicz.gov.pl; 7 Institute of Biomedical Engineering, University of Silesia in Katowice, 39 Będzińska Str., 41-200 Sosnowiec, Poland; pawel.popielski@us.edu.pl; 8 Materials Research Laboratory, Faculty of Mechanical Engineering, Silesian University of Technology, 18a Konarskiego Str., 41-100 Gliwice, Poland

**Keywords:** ethylene–vinyl acetate (EVA), silver, particles, composites, antimicrobial, *Candida*, mechanical properties, wettability, cytotoxicity

## Abstract

Ethylene–vinyl acetate (EVA) is a versatile polymer for applications in dental devices; however, its vulnerability to microbial colonization increases with long-term use. This study evaluates EVA composites modified with silver–sodium–hydrogen–zirconium phosphate (SP) particles, aimed at enhancing antimicrobial performance while preserving key functional properties. Composites containing 1–16 wt.% SP were prepared via solvent-based and mechanical compounding routes, with scanning electron microscopy confirming correct filler distribution across processing methods. Antimicrobial assays revealed a pronounced reduction in *Streptococcus mutans* and *Candida albicans* levels, reaching 88% and 98% antimicrobial efficacy, respectively, at 16 wt.% SP. Cytotoxicity testing with L-929 fibroblasts demonstrated maintained cell viability above the 70% threshold, confirming non-cytotoxicity. Mechanical characterization indicated marginal increases in hardness, slight tensile strength reduction at higher filler loadings, while other physicochemical and thermal analyses showed minimal impact on polymer performance. These findings indicate balanced antimicrobial activity with other biofunctional properties.

## 1. Introduction

Copolymers of ethylene with vinyl acetate (EVA), due to their favorable combination of low hardness, elasticity, impact energy dissipation, ease of thermoforming, and biocompatibility, are widely used in the manufacturing of intraoral devices such as occlusal splints, orthodontic retainers, and sports mouthguards [1,2,3]. This thermoplastic and semicrystalline copolymer is composed of ethylene with vinyl acetate (usually up to 40% by weight) monomers [4]. Depending on the intended production method, EVA is supplied as granules, rolled sheets, or pre-formed semi-finished products that require only final shaping [5,6,7]. The material properties and degree of crystallinity are strongly influenced by the ratio of ethylene to vinyl acetate (VA) monomers in the macromolecular structure. This offers significant potential for tailoring material characteristics. For instance, a polymer containing 9% VA exhibits a crystallinity of 46% and a Young’s modulus of 118 MPa, whereas a polymer with 40% VA shows a crystallinity of only 8% and a modulus of 3.4 MPa [8]. Thermal properties, such as melting temperature—which are crucial for processing behavior—are also strongly dependent on the VA content. Experimentally, the melting temperatures for the aforementioned materials are 102 °C and 45 °C, respectively [8]. An increase in vinyl acetate (VA) content also leads to a reduction in tensile strength [9] and an increase in water sorption [10], despite the overall hydrophobic nature of the material [11]. Although EVA exhibits unique characteristics that allow for versatile intraoral applications, several limitations must be taken into account. These include difficulties in achieving optimal thickness during thermoforming [12,13], as well as a progressive loss of thickness over time, which compromises its ability to dampen forces and, consequently, reduces the protection it provides to oral tissues against trauma [14]. Additionally, temperature fluctuations and repeated pressure changes during the routine use of EVA-made devices can affect the crystallinity of materials, leading to hardening and a reduction in their protective ability [15]. Another important concern is the time-dependent alteration of surface morphology, which promotes microbial accumulation by making effective surface cleaning more difficult [16,17]. Improper storage combined with imperfect hygiene may cause the devices themselves to become a reservoir of microorganisms over time [18,19], which may reduce their lifespan and adversely affect the user’s health [17]. Polymeric materials exposed to the oral environment tend to undergo unfavorable changes over time, such as increased surface roughness and microcrack formation, which compromise hygienic maintenance [20,21]. Examples of consequences of this include the fact that colonization of polymer orthodontic overlays may contribute to gingival recession and periodontal problems [22], and the use of thermoplastic retainers promotes colonization of tooth surfaces by cariogenic bacteria *Streptococcus mutans* and *Lactobacillus* spp. [23]. The introduction of polymeric orthodontic devices into the oral cavity also promotes the increased presence of pathogenic yeast-like fungi [24] in both adult patients [24] and children [25].

In the case of non-medical products such as mouthguards for athletes, the situation becomes even more complicated. Frequent removal of the mouthguard and its direct exposure to the external environment facilitate the introduction of additional microorganisms. As a result, beyond the typical bacteria and yeasts inhabiting the oral cavity, other microbes—such as soil-dwelling organisms (e.g., *Rhodotorula*, *Bacillus cereus* in football players; *Pseudomonas* spp. in hockey players)—may also colonize the surface. Furthermore, colonization by *Candida* spp. and *Staphylococcus aureus* is often increased [26]. This problem also occurs in children using mouthguards, where numerous microorganisms—typically found in soil, plants, or aquatic environments—are present, such as *Stenotrophomonas maltophilia*, *Pseudomonas putida*, *Microbacterium* spp., *Granulicatella adiacens*, *Staphylococcus pasteuri*, *Staphylococcus xylosus*, *Staphylococuss saprophyticus*, *Staphylococcus warneri*, *Pseudomonas monteilii*, *Enterobacter ludwigii*, *Enterobacter cloacae*, *Bergeyella zoohelcum*, *Chryseobacterium culicis* [17]. Over time, the degree of microbial colonization of the mouthguards increase, leading to an intensification in the frequency of oral lesions [27,28]. Moreover, many children lose motivation to maintain proper hygiene of their devices and oral cavity [28], which is particularly dangerous, considering that storing mouthguards in a humid and closed environment increases the possibility of long-term survival of, among others, cariogenic bacteria [29]. Neither mechanical nor chemical cleaning methods for EVA devices are fully effective [30,31,32]. Chemical agents strongly reduce colonies, but do not remove all [30,33], while mechanical cleaning using soft brushes creates microscopic scratches on the surface, which facilitate recolonization and make subsequent chemical disinfections more difficult [34]. The above considerations suggest that it is reasonable to modify EVA to impart antimicrobial properties. Despite its importance, research in this area is relatively scarce. Silver nanoparticles are used for surface modification and show effective reduction in *Streptococcus sobrinus*, *Porphyromonas gingivalis*, *Staphylococcus aureus,* and *Escherichia coli* levels [18,35]. Polyethylene-co-vinyl acetate compounded with silver-calcined scallop shell powder [36], oyster shell powder [37], and zinc oxide [38] also shows antibacterial effect against *Staphylococcus aureus* and *Escherichia coli*. EVA coated with chlorhexidine hexametaphosphate nanoparticles prevented the growth of *Pseudomonas aeruginosa* and *Staphylococcus aureus* [39]. Glass ionomer particles emitting F^-^ ions into the environment were also used, which resulted in a bacteriostatic effect on *Streptococcus mutans* (*S. mutans*) and *Porphyromonas gingivalis* and inhibited the formation of *S. mutans* biofilm [40]. Most of these materials were characterized by impaired mechanical properties, and their potential cytotoxicity remains unknown.

To gain further insight, it is necessary to conduct studies using novel experimental materials. This article aims to explore the property relationships of EVA-based composites enriched with varying concentrations of silver sodium hydrogen zirconium phosphate (SP). The research hypothesis assumes that incorporating SP into experimental EVA-based composites—intended for use in intraoral appliances—will reduce the adhesion of pathogenic yeast-like fungi and cariogenic bacteria on the material surface, while preserving the desired biofunctional properties.

## 2. Materials and Methods

### 2.1. Composites Preparation

#### 2.1.1. Materials

Starting materials were EVA EVATANE® 28–40 (SK Functional Polymer, Puteaux, France) containing 28% of vinyl acetate and characterized by hardness 73 Shore A (a Melt Flow Index at 190 °C, 2.16 kg is 35–45 g/10 min), used as a matrix and silver-sodium-hydrogen-zirconium phosphate (SP) with a summary formula Ag_0.46_Na_0.29_H_0.25_Zr_2_(PO_4_)_3_ (Milliken Chemical, Spartanburg, SC, USA) in form of submicron particle size powder containing 10% (*w*/*w*) silver, which was used as a filler [41].

#### 2.1.2. Composites Preparation

The solvent method was chosen as the primary method for incorporating filler into the experimental materials. Method development began with an experiment involving polymer solubility tests in seven selected solvents: chloroform (trichloromethane) 98.5% (Chempur, Piekary Śląskie, Poland), dichloromethane 99% (PureLand, Kraków, Poland), triethyl tetraamine min. 97% (Sigma-Aldrich, St. Louis, MO, USA), 1,2,4-trichlorobenzene 99% (Acros Organics, Geel, Belgium), cyclohexane 99% (Acros Organics, Geel, Belgium), xylene 99% (Acros Organics, Geel, Belgium), and toluene (methylbenzene) 99% (Acros Organics, Geel, Belgium).

Tests were performed using standardized volumes of each solvent (20 mL), to which 1 g of EVA was added. The solvent containing the material sample was placed in a revolver and mixed at 150 rpm with a sudden deceleration every 30 s. The experiment was conducted at room temperature in an air-conditioned room. After 2 h, the system was checked organoleptically. If the entire polymer mass had dissolved, another 1 g was added. Otherwise, the solvent was eliminated from further experiments. The procedure was repeated until a total of 4 g of EVA had been added (higher concentrations were not used due to the significantly increased viscosity of the system). The best solubility results were obtained for chloroform and toluene, and chloroform was selected for further testing due to the improved flowability of the resulting system, which allowed for easy manipulation during further work.

The possibility of dissolving the polymer on a larger scale was then confirmed in an experiment conducted in a 1000 mL pharmacy jar, to which 500 mL of chloroform and 100 g of EVA granulate were added. The process was conducted for 4 h at 21 ± 1 °C temperature using Hei-TORQUE Ultimate 100 stirrer (Wood Dale, IL, USA) at 150 rpm, which allows the granules to dissolve completely. A procedure for solvent evaporation was then developed. In the first stage, the solvent was preliminarily evaporated under a reduced pressure of 100 mbar on a rotary evaporator for 20 min at 50 °C (IKA^®^ HB10 digital, Guangzhou, China); however, the possibility of it remaining freely pouring was still retained. Afterward, the material was poured into Petri dishes with a diameter of 120 mm and a height of 20 mm, and evaporation was continued for 12-h cycles in laboratory dryers at 65 °C (above the boiling point of chloroform, 61.2 °C, and below the polymer’s melting point, 70 °C). To confirm the effectiveness of the process, FTIR analyses were performed on samples taken from cross-sections of the material. FTIR studies were performed using a Spectrum Two spectrometer (Perkin-Elmer, Waltham, MA, USA)—128 samples scanned with a resolution of 1 cm^−1^. Characteristic chloroform absorption bands were searched for at maximum ~3019 cm^−1^ (C–H stretching) and ~755 cm^−1^ (C–Cl stretching) [42]. The absorption band from C–H-bending at 1215 cm^−1^ was not taken into account due to similarity to the absorption band from C–O stretching of EVA. The presence of chloroform was not confirmed after 24 h.

Finally, the composites were developed by incorporating SP into chloroform-solubilized EVA at concentrations of 1%, 2%, 4%, 8%, and 16% (composite abbreviations as EVA-C1, EVA-C2, EVA-C4, EVA-C8, and EVA-C16). The system was initially mechanically mixed for approximately 1 min at 700 rpm using a magnetic stirrer to distribute the filler throughout the suspension, followed by two 5-min ultrasonic homogenization cycles using a Sonic Ruptor 250 homogenizer (Omni International, The Homogenizer Company, Kennesaw, GA, USA)—80% pulsation, 90% power—while continuing mixing on the magnetic stirrer. The entire process was conducted at 60 °C. Next, the previously developed solvent evaporation procedure was applied, confirming its effectiveness with FTIR tests, which were necessary since the filler affects the viscosity of the system, which may result in a longer evaporation time.

Mechanical compounding was performed with a mechanical homogenizer, Zamak Mercator (Zamak Mercator, Skawina, Poland), with a process divided into three steps. The entire process was conducted at 100 °C. The first phase involved mixing for 5 min at 10 rpm, the second for 5 min at 30 rpm, and the third for 1 min at 10 rpm, after which the material was removed. The filler was introduced in the first step, gradually adding it to the granulate. With this method, two materials were obtained with concentration SP of 1% (EVA-M1) and 16% (EVA-M16).

The test samples were made on a Roko Multipress Pro injection molding machine (ROKO, Częstochowa, Poland) in the form of ready-to-use sheets of a specific thickness, from which the samples were cut. All samples were visually analyzed to eliminate samples with bubbles or other imperfections.

The qualitative assessment of filler dispersion in the matrix after both methods of filler introduction was performed using a scanning electron microscope (Supra 25, Zeiss, Jena, Germany). Fractures (cross-sections) obtained by breaking samples cut on both sides to approximately ¼ thickness in liquid nitrogen were analyzed. All samples were sputtered with gold. The accelerating voltage ranged from 1 to 5 kV.

### 2.2. Antimicrobial and Cytotoxicity Tests

Samples measuring 10 × 10 mm and 2 mm thick were sterilized at the Medical Device Radiation Sterilization Station in Warsaw using an electron beam with an energy of 10 MeV and an average power of 10 kW. Additionally, the sterilization of hardness test samples prepared for this purpose was carried out, and tests showed that the sterilization process did not cause any changes in hardness.

#### 2.2.1. Antimicrobial Properties

The samples (five from each material) were placed in 1 mL of a suspension of the reference strain *Candida albicans* ATCC 10231 (*C. albicans*) or *S. mutans* ATCC 33535 (*S. mutans*) with a final density of 1.5 × 10^5^ CFU/mL in tryptone water. After 17 h of incubation at 37 °C under dynamic conditions, a volume of 20 μL of the suspension was plated on a solid culture medium: Sabouraud agar (bioMerieux, Marcy l’Etoille, France) for *C. albicans* or Columbia agar with 5% sheep blood plates (bioMerieux, Marcy l’Etoille, France) for *S. mutans*. After 17 h of incubation at 37 °C, the number of colony-forming units (CFU) was counted with a colony counter (ProtoCOL 3 PLUS, Synbiosis, Frederick, MD, USA), and antimicrobial efficiency (AME) was calculated as the percentage reduction in the number of microorganisms compared to the positive control [43].

#### 2.2.2. Cytotoxicity—MTT Assay

The cell viability assay was performed based on the EN ISO 10993-5:2009 [44] standard with previously described details [45]. Samples (six from each material) were placed separately in 2 mL of culture medium used for the culture of fibroblasts of the L-929 line and incubated at 37 °C in an atmosphere of 5% CO_2_ for 2 or 10 days. Under the same conditions, a control (the culture medium) was incubated. A suspension of cell culture of the L-929 line (NCTC clone 929, American Type Culture Collection, catalog number CCL-1, Manassas, VA, USA) with a final density of 1 × 10^5^ cells/mL of medium was used. Mouse fibroblasts were incubated under in vitro culture conditions for 24 h with undiluted extracts, and their viability was assessed using the MTT assay. To extract MTT formazan, DMSO was used. The Eon automatic plate reader (BioTek Instruments, Winooski, VT, USA) was used to measure the absorbance at 550 nm, and the percentage of cell viability was calculated as a relation of the absorbance of the test sample to the absorbance of the control.

### 2.3. Physico-Mechanical Properties

#### 2.3.1. Shore A Hardness Measurements

The Shore A hardness was tested in accordance with the ISO 868 standard [46]. Three samples were made from each material (40 mm in diameter and 6 mm high). Five indentations with a time of 5 s at a temperature of 21 ± 1 °C were performed on each sample using a Bareiss HPE II-A (Bareiss, Oberdischingen, Germany) durometer.

#### 2.3.2. Tensile Test

Five samples of type 5B specified by the EN ISO 527-2 standard [47] of each material were cut from 2 mm thick plates. Samples were tensile tested at a crosshead speed of 10 mm/min until the break using a universal testing machine (Zwick Z020, Zwick GmbH & Co., Ulm, Germany), and the ultimate tensile strength *TS* (MPa) was calculated as the ratio of force at rupture (N) and the initial cross-sectional area (mm^2^). Toughness (MJ/mm^2^) was calculated as the total area under the stress–strain curve up to the point of fracture.

#### 2.3.3. Differential Scanning Calorimetry (DSC)

Differential Scanning Calorimetry (DSC) measurements were performed in accordance with ISO 11357-1:2023 and ISO 11357-3:2018 standards [48,49] using the DSC 3 (Mettler Toledo, Greifensee, Switzerland). Samples (five from each material) weighing approximately 2.5 mg were placed in aluminum crucibles, and a heating rate of 10 K/min in the air was used. First run (heating) was in the temperature range of −20° C to 200° C (stopping for 5 min at 200° C), second was cooling to the temperature of −20 °C (stopping for 5 min at −20° C), third was heating to the temperature of 280 °C. The melting temperature T_m_, considered as the temperature at which the endothermic peak occurred, was determined using Mettler Toledo STAR SW 16.30 (Mettler Toledo, Greifensee, Switzerland) software.

#### 2.3.4. Water Contact Angle (CA)

The water contact angle was tested. Teen samples were made from each material. Deionized water (4 µL) was dropped onto the tested surface of the samples (20 mm × 20 mm × 2 mm) using the sessile drop method. A goniometer (OCA 15EC, Data Physics, Filderstadt, Germany) was used.

#### 2.3.5. Dynamic Mechanical Analysis (DMA)

Cylindrical samples measuring 2 ± 0.05 mm in height and 5 mm in diameter were used. The tests were conducted using a Mettler-Toledo DMA/SDTA 861e device (Mettler-Toledo GmbH, Schwerzenbach, Switzerland). The measurement was performed in shear mode at a temperature range from −150 to 100 ± 0.5 °C, with a force amplitude of 14 N, a displacement amplitude of 1 μm, and a frequency of 1 Hz. The heating rate was 3 °C/min. The storage modulus G′ and loss modulus G″ for the samples were determined, as well as the mechanical loss factor tgδ (the amount of energy dissipated during the sample loading and unloading cycle), which was the ratio of G″ to G′ [50].

### 2.4. Statistical Analysis

The results were analyzed using a one-way analysis of variance (ANOVA, α = 0.05). Normality was verified using the Shapiro–Wilk test (*p* > 0.05). Homogeneity of variance was verified using Levene’s test (*p* > 0.05). If the assumption of equal variances was not met, Welch’s F correction was used. If the null hypothesis was rejected, the Tukey HSD post hoc test was used. For small sample sizes (antimicrobial efficacy), the nonparametric Kruskal–Wallis test (α = 0.05) was used. Analyses were performed using Statistica version 13.1 (TIBCO Software Inc., San Ramon, CA, USA).

## 3. Results

### 3.1. Solvent Evaporation Assessment

**Figure 1:**
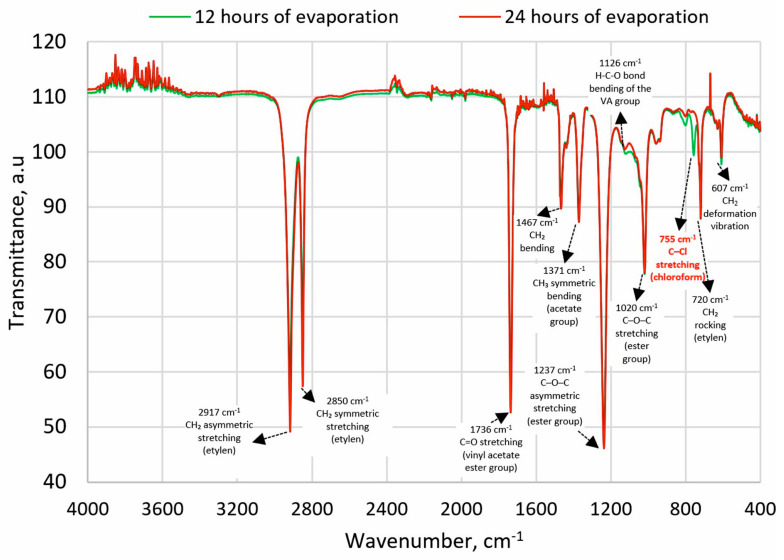
FTIR spectra of EVA after 12 h and 24 h of solvent evaporation; description based on [51,52].

The FTIR spectra were analyzed for characteristic absorption bands of chloroform with a maximum at approximately 3019 cm^−1^ (stretching C-H) and 750 cm^−1^ (stretching C-Cl) [42]. After 12 h of evaporation, the first absorption band was not observed, but the second was detected (Figure 1). After the next 12 h, no absorption bands characteristic of chloroform were detected, confirming that the solvent had evaporated to a level below its detection limit.

### 3.2. Distribution of Filler in the Matrix

**Figure 2:**
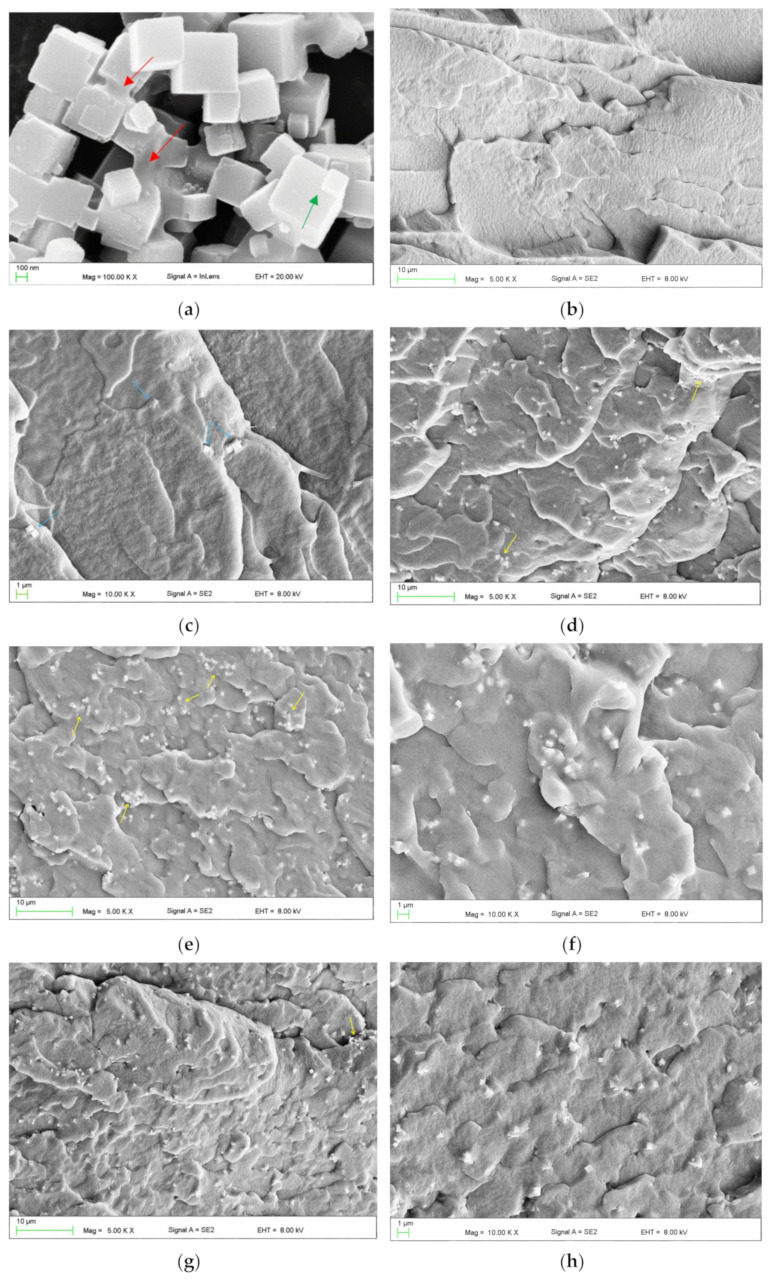
Representative SEM microphotographs presenting morphologies of SP powder (**a**), frozen-fractured cross-sectional area of control material EVA (**b**), EVA-C1 (**c**), EVA-C8 (**d**), EVA-C16 (**e**,**f**), and EVA-M16 (**g**,**h**).

A representative SEM micrograph illustrating the morphology of the filler particles is presented in Figure 2a. Characteristic cubic structures were observed, with particle sizes ranging from approximately 100 to 600 nm. Additionally, larger structures were identified, with linear dimensions in the selected direction often exceeding 1 μm. These included distinct, permanently connected cubic forms (indicated by green arrows) and microparticles with more irregular shapes (indicated by red arrows). Figure 2c–f present representative micrographs of the cross-sectional morphology of frozen-fractured samples. At the lowest concentration, the presence of up to several particles or their clusters/aggregations (indicated by blue arrows) was observed in each observation field (Figure 2c). With increasing filler concentration, the reduction in the distance between adjacent particles/aggregations occurred. It was typically between 5 and 20 µm for EVA-C1/EVA-M1, and 4 and 8 µm for EVA-C16/EVA-M16. A trend toward an increase in the aggregation size was also observed. The presence of single cubic particles was rare. In contrast, clusters/aggregations of up to a few cubic particles were the dominant form (Figure 2d,e,g), although it is difficult to say with certainty if their presence was due to the quality of the filler distribution or related to the morphology of the powder used. Starting from the EVA-C4 material, much larger aggregations were observed, indicated by yellow arrows. Their sizes ranged from 2 μm to 8 μm, with significantly more observed in the case of EVA-C16 than for EVA-M16, which indicates better effectiveness of mechanical compounding (Figure 2e–h). The results confirmed the expected filler distribution in the matrix.

### 3.3. Antimicrobial Properties

**Figure 3:**
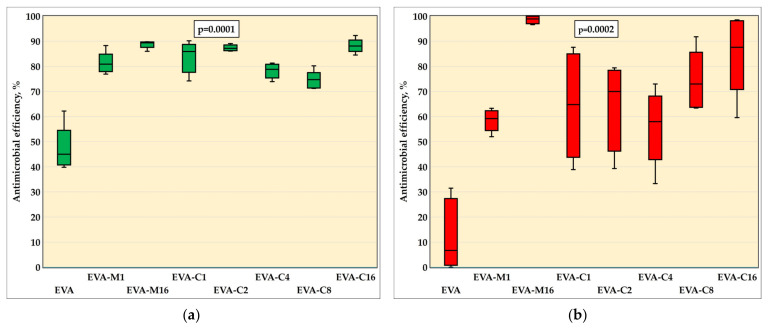
Antimicrobial efficiency (medians, interquartile ranges, minimum value, and maximum values) against *S. mutans* ATCC 33535 (**a**) and *C. albicans* ATCC 10231 (**b**) strains with Kruskal–Wallis test results (α = 0.05).

**Figure 4:**
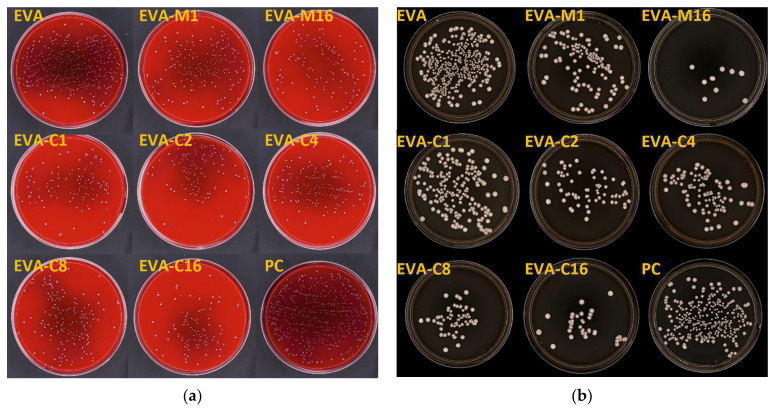
Representative images of cultured plates after incubation with the *S. mutans* ATCC 33535 (**a**) and *C. albicans* ATCC 10231 (**b**) suspensions.

The results of the AME test against the *S. mutans* (ATCC 33535) strain are in Figure 3a and in Table 1, while Figure 4 presents sample photographs of plates incubated with the test suspension. The differences between the medians were statistically significant (*p* = 0.0001). The composites with the highest filler content, EVA-M16 and EVA-C16, demonstrated the highest AME values, with medians of 89.4% and 88.1%, respectively. However, the values observed for all composites were similar. For EVA, the AME median was 45.0%. At the same time, the median number of cells that survived in suspension with the EVA material was from above two times to above five times higher than for composites (Table 1).

**Figure 5:**
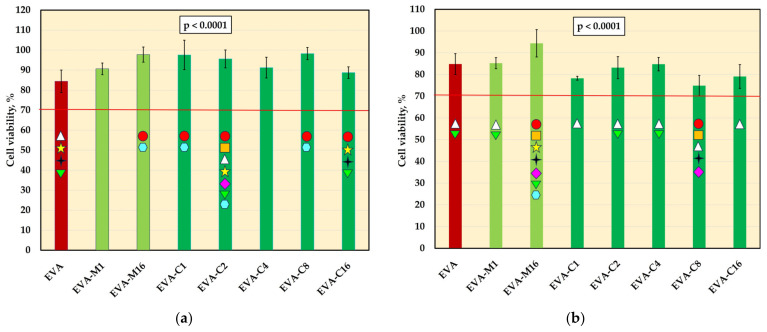
The viability of L-929 cells (mean values and standard deviations) after incubation with 2-day (**a**) and 10-day (**b**) extracts; values below the 70% (red-line) threshold indicate cytotoxicity in accordance with ISO 10993-5:2009; *p*-values are one-way ANOVA test results (α = 0.05), subsequent materials are marked on the axis with colored symbols; the symbol on the bar means that the average value was statistically significantly (Tukey’s HSD test, α = 0.05) different in comparison with the value obtained for the material marked with these symbols on the axis.

The results of the AME test against the *C. albicans* are presented in Figure 3b and in Table 1, while Figure 5 presents sample photographs of plates incubated with the test suspension. The differences between the medians were statistically significant (*p* = 0.0002). The composites with the highest filler content, EVA-M16 and EVA-C16, showed the highest AME values, with medians of 98.9% and 87.6%, respectively. The median values observed for composites characterized with lower SP content ranged from 58% (EVA-C4) to 73% (EVA-C8); however, large values of IQR and differences between minimum and maximum values were noted. For EVA, the AME median was 6.7%. At the same time, the median number of cells survived in suspension with the EVA material was from above two times to above seven and a half times higher than for composites (Table 1).

### 3.4. Cytotoxicity Test

The mean viability values of L-929 cells are presented in Figure 5. For 2-day extracts, significant differences were observed for the control material (84.5%) and five composites, and the mean viabilities were generally higher. None of the experimental material extracts showed decreased cell viability compared to EVA, and all average values remained above 70%. For 10-day extracts, significant differences were also observed. However, the post hoc test confirmed a statistically significant decrease in viability compared to EVA only for EVA-C8, which showed the lowest mean value (74.3%), with one individual value (69.1%) falling below 70%. Student’s t-test revealed a significant reduction (*p* < 0.05) in viability after prolonged extraction for most materials, except EVA and EVA-M16. In the case of EVA-M16, a reduction in viability was observed, but it was not statistically significant due to high standard deviations.

### 3.5. Mechanical Properties

**Figure 6:**
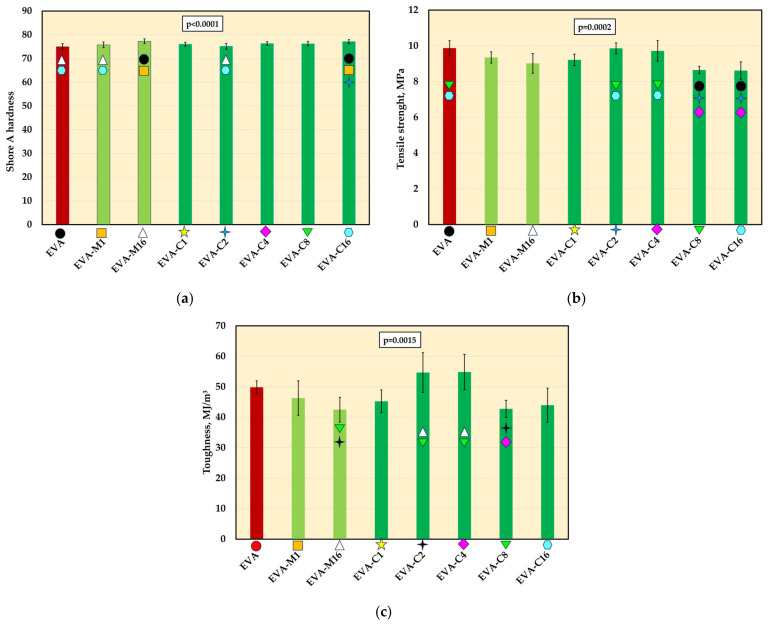
The Shore A hardness (mean values and standard deviations) of EVA and experimental composites (**a**), tensile strength (**b**) and toughens (**c**); *p*-values are one-way ANOVA test results (α = 0.05), subsequent materials are marked on the axis with colored symbols; the symbol on the bar means that the average value was statistically significantly (Tukey’s HSD test, α = 0.05) different in comparison with the value obtained for the material marked with these symbols on the axis.

The mean Shore A hardness values are presented in Figure 6a. A significant increase in hardness was observed for the composites with the highest filler concentration (EVA-M16 and EVA-C16) compared to the neat polymer. Additionally, the hardness of EVA-M1 and EVA-C2 was significantly lower than that of EVA-C16. No significant differences were found between materials with the same filler concentration introduced using different methods. The mean hardness values ranged from 75 Shore A (EVA) to 77.3 Shore A (EVA-M16).

Tensile strength values (Figure 6b), compared to EVA (9.9 MPa), decreased for most of the experimental composites. However, significantly lower mean values were recorded only for EVA-C8 and EVA-C16 (both 8.6 MPa). For the EVA-M16 composite, the mean tensile strength was 9.0 MPa. For the remaining composites, the mean strength values ranged from 9.2 MPa (EVA-C1) to 9.9 MPa (EVA-C2).

Significant differences in toughness (Figure 6c) were observed only between the composites themselves, but not in comparison to the neat EVA polymer.

The DMA test results are presented in Table 2. The G′ values for the composites at 23 °C ranged from 6.22 MPa for EVA-C1 to 8.68 MPa for EVA-C16, and for EVA, it was 7.19 MPa. The storage modulus values were from 36% (EVA and EVA-C8) to 39% (EVA-C4) lower at 37 °C. The G″ values at 23 °C for EVA were 0.56 MPa, while for composts ranged from 0.40 MPa (EVA-C1) to 0.62 MPa (EVA-C16). The loss modulus values were from 20% (EVA-C1) to 23% (EVA-C16) lower at 37 °C. The tanδ values were 0.07 (all materials) at 23 °C, and at 37 °C they ranged from 0.8 to 0.09 (EVA).

### 3.6. Contact Angle

**Figure 7:**
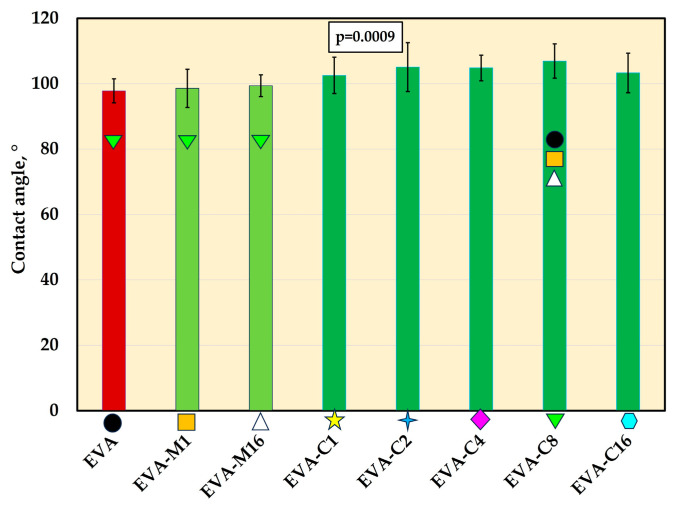
The contact angle (mean values and standard deviations) of EVA and experimental composites, *p*-values are one-way ANOVA test results (α = 0.05), subsequent materials are marked on the axis with colored symbols; the symbol on the bar means that the average value was statistically significantly (Tukey’s HSD test, α = 0.05) different in comparison with the value obtained for the material marked with these symbols on the axis.

**Figure 8:**
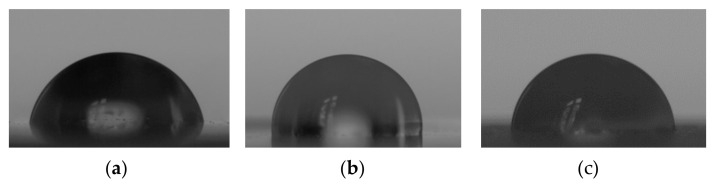
Representative images of deionized water droplets on the surfaces of EVA (**a**), EVA-M16 (**b**), and EVA-C16 (**c**) obtained from the goniometry camera.

For all materials, contact angle mean values were above 97° (Figure 7 and Figure 8). The composites exhibited higher contact angle values than EVA, but the differences were statistically significant (*p* ≤ 0.05) only for EVA-C8 (mean value of 107.9°). The contact angle value for EVA-C8 was also significantly higher than for EVA-M1 (98.6°) and EVA-M16 (99.4°); however, the *p*-values were 0.02 and 0.04, respectively, which were close to the *α*-value.

### 3.7. Melting Temperature (DSC)

**Figure 9:**
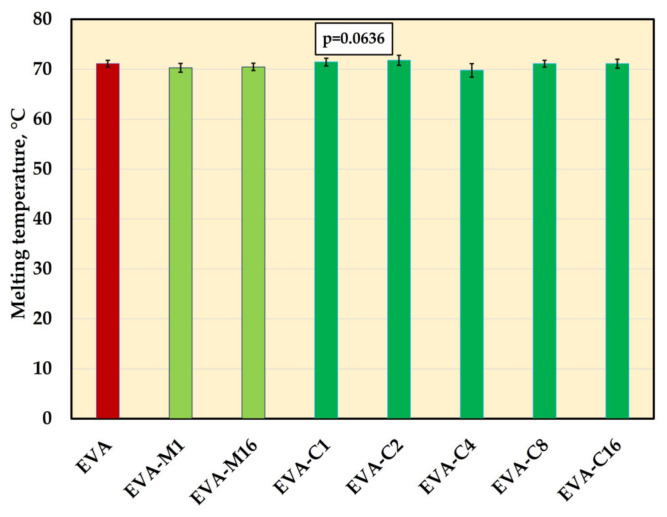
The melting temperature (mean values and standard deviations) of EVA and experimental composites, *p*-values are the one-way ANOVA test result (α = 0.05).

The significant changes in melting temperature mean values were not registered (Figure 9, Supplementary Figure S1). The values ranged from 69.7 °C (EVA-C4) to 71.8 °C (EVA-C2).

## 4. Discussion

The design of EVA medical devices intended for use in the oral cavity results in the formation of a closed microenvironment during several hours of daily application. The surface in contact with the teeth and oral mucosa is exposed to 100% humidity and elevated temperature, while the device simultaneously prevents natural self-cleaning by saliva. This environment promotes the colonization of both materials and oral tissues by pathogenic microorganisms, such as yeast-like fungi [24] and cariogenic bacteria [23].

Clinical studies on removable orthodontic appliances, retainers, and occlusal splints confirm these findings [23,25,53]. Significant difficulties in maintaining proper hygiene are indicated, which increase with the time of exploitation [17,18], which, in addition to the oral environment, is influenced by the methods of cleaning [53], storing products [29], and their manufacturing technology [54].

Despite a number of experiments conducted to develop EVA characterized by increased antimicrobial resistance, this problem remains relatively poorly recognized, especially compared to the wide knowledge regarding other dental materials, such as composite resins or denture polymethacrylate resins [55,56,57]. The potential use of silver in this area has long been highlighted; however, attention has been focused on nanoparticles [18,35], which have proven activity against cariogenic bacteria and yeast-like fungi [58], while more complex materials were not considered. The filler used in the current work, silver hydrogen zirconium phosphate, exhibits antimicrobial action activated under wet conditions and based on the exchange of cations such as Na^+^ or Ca^2+^ from the environment with silver ions from the inorganic and insoluble carrier [59]. Silver ions also play a fundamental role in the antimicrobial activity of silver nanoparticles, which can continuously release these ions over time. This action is widely described in review works [60]. Due to their electrostatic attraction and strong affinity for sulfur-containing proteins, silver ions can attach to the bacterial cell wall and cytoplasmic membrane. This adhesion increases membrane permeability and can ultimately disrupt the integrity of the bacterial envelope. Once inside the cell, free silver ions can deactivate respiratory enzymes, leading to the generation of reactive oxygen species and inhibition of adenosine triphosphate production. The resulting reactive oxygen acts as key agent in damaging the cell membrane and modifying DNA. Since sulfur and phosphorus are essential components of DNA, the interaction between silver ions and these elements can interfere with DNA replication and cell division, potentially causing cell death. Additionally, silver ions can inhibit protein synthesis by denaturing ribosomes in the cytoplasm, further contributing to the antimicrobial effect. Nanoparticles themselves also show antimicrobial properties. Their accumulation can cause denaturation of the cell membrane, while their nanoscale size enables them to penetrate bacterial cell walls and alter membrane structure, which can lead to organelle rupture and cell lysis. Furthermore, they can disrupt the transduction signal, leading to cell apoptosis and termination of cell multiplication. The effect in the case of nanoparticles depends on many factors, such as their size, shape, dispersion, and surrounding media properties [61]. As mentioned earlier, the filler exhibits its antimicrobial effect only through the release of ions, but composites based on polymethyl methacrylate during in vitro experiments have demonstrated at least ninety days of continuous and stable antimicrobial activity in an aqueous environment [62], while with nanosilver the effect is relatively quickly limited or even stopped [63,64]. The larger particle size also facilitates efficient incorporation of the filler into the matrix [65]. Another advantageous property of SP, particularly relevant to EVA materials used in dental and sports applications where esthetics are critical, is the absence of the dark brown discoloration typically associated with nanosilver [66,67]. Although the composites became slightly whitish and less transparent, this effect is acceptable, as it does not interfere with subsequent color modification using dyes commonly applied to EVA to achieve the desired shade.

The mechanical method employing a homogenizer was relatively straightforward to implement; however, it permitted the preparation of only limited composite quantities (29 g per batch) and required stringent process hygiene, particularly with respect to equipment cleanliness between different batches and material types. These factors contribute to the method’s high labor intensity. In contrast, the solvent-based approach enabled the production of 100 g of composite in a single, multi-step procedure. Despite the total processing time of approximately 24 h, the method is characterized by relatively low labor and time demands. Due to this reason, only two concentrations (the lowest and the highest) were selected for mechanical mixing to compare the results with the solvent method. However, it was assumed that if significant differences in dispersion were observed during SEM tests, intermediate concentrations would also be developed. It should be noted, however, that for larger-scale composite production, mechanical compounding is likely to be more appropriate, given the ecological and economic considerations that justify the need for the creation of a solvent recovery system. SEM studies showed that both methods achieved qualitatively comparable filler dispersion on the analyzed scale. Usually, numerous relatively small aggregations were observed, which in many cases could be permanently bonded particles observed in the powder itself. Nevertheless, the material with the highest filler content, modified via the solvent method, exhibited a significantly greater tendency to form large aggregates (several micrometers in size) compared to the composite modified by mechanical compounding. Despite this, the filler dispersion in all analyzed cases can be described as satisfactory.

The AME values of the composites against the *S. mutans* strain were comparable for all materials, with significantly higher values than reported for EVA. A similar situation was observed for *C. albicans*, although for EVA-M16, a higher median and smaller interquartile range values were registered in comparison to EVA-C16, which can be related to the previously mentioned smaller number of aggregations, because more uniform dispersion increases the surface area of particles that can release silver ions into the environment [68,69]. At the same time, it was observed that the greatest increase in antimicrobial effectiveness occurred with the addition of 1% filler. Beyond this concentration, the activity remained stable against bacteria, and for yeast-like fungi, it either continued to increase at a significantly lower rate or plateaued. These findings are consistent with other studies on polymer composite materials, such as EVA/zinc oxide nanocomposites [70], PVA composites containing silver and zinc oxide nanoparticles [71], or silicone elastomer enriched with silver-releasing ceramic carrier [72]. In addition to the reduction in effective surface area observed at higher filler loadings, another factor that may contribute to the limited increase in antimicrobial efficacy is the diffusion resistance within the elastomeric polymer matrix. The structure of the polymer can significantly hinder the movement of antimicrobial agents from the interior of the matrix to its surface. As a result, even when the concentration of antimicrobial filler is increased, the actual amount of active substance released into the surrounding environment may remain limited because this diffusion barrier slows down the migration of antimicrobial ions, preventing them from reaching the surface [73,74,75]. Therefore, the antimicrobial action does not increase proportionally with filler content. Attention should be focused on the reduction in CFU/mL numbers for composites compared to EVA, which for *S. mutans* was approximately two-fold, and for *C. albicans*, even over seven and a half times higher than for the unmodified polymer. This data better illustrates the differences, considering that pure EVA also exhibited antimicrobial activity. The observed microorganism reduction in the microenvironment containing the control samples may result from the release of leached hydrocarbons and compounds containing hydroxyl and carbonyl functional groups, such as alcohols, lactones, esters, and ketones, which occur in small amounts in these materials [76]. It should be emphasized that there are no reports indicating that the release of ingredients from EVA inhibits the growth of microorganisms during the daily use of mouthguards or orthodontic devices. However, laboratory studies have shown that polymer-based dental materials can exhibit similar effects for a short period after sample preparation [77,78]. This antimicrobial activity appears to be mild and related to the structural characteristics of microbial cells. Significantly higher AME values were observed for EVA in the case of the sensitive S. mutans, whereas for yeast-like fungi, the average AME was 6.7%. In one sample, the value of 9.15 CFU/mL was lower than that of the positive control. These differences can be explained by considering that the *C. albicans* cell wall is composed mostly of carbohydrates. Moreover, the microfibrillar polymers, such as β-glucans and chitin, represent the structural components of the wall that form a rigid skeleton that provides strong physical properties to the cell and protects the cell against the mentioned ingredients; *S. mutans* bacterium has a relatively simple cell structure, a thin and porous cell wall (peptidoglycan), and a simpler membrane system [79,80,81]. In this study, a substantial reduction in the number of microorganisms was achieved; however, even at the highest concentrations, complete elimination was not observed. The pronounced activity against *C. albicans* is advantageous in light of the frequent complications associated with its presence [82], and no less important is the activity against *S. mutans*, given the widespread occurrence of this bacterium on EVA products used by both children and adults [17,28]. It is worth emphasizing that the antimicrobial properties of both silver ions and silver nanoparticles are well established [83]. Nevertheless, reports addressing their incorporation into EVA remain scarce [18,84] and, to date, no studies have described EVA modification employing ceramic-based silver carriers. Improving the properties of EVA in this respect is particularly important, given that it is most commonly used for the fabrication of orthodontic appliances and mouthguards, with children and athletes (including children) being primary users. It should be noted that, despite the application of mechanical and chemical cleaning methods using commercially available agents intended for this purpose [34], there are no established standards for mouthguard hygiene [19]. Moreover, only about 3% of users perform disinfection [4], which, in turn, may lead to material hardening [4]. However, the limitations resulting from the applied methodology and the outcomes obtained should be taken into account. It is important to note that the conducted in vitro studies do not fully replicate real-life conditions, either in terms of the microbial environment composition or the growth of microorganisms in the form of biofilms—particularly multispecies biofilms [85,86]. Moreover, under physiological conditions, the preadsorption of salivary proteins onto material surfaces, which can modulate the initial attachment of microorganisms, may also limit the release of silver ions, thereby progressively diminishing or even fully suppressing the antifungal effect. On the other hand, the choice of methodology was justified by the need to examine the reduction potential of strictly defined microorganisms (each considered individually) under controlled, reproducible laboratory conditions, which provides a starting point for further, more comprehensive experiments.

Because the toxicity of silver ions has been proven [87,88] to indicate the potential hazards associated with SP introduction, cytotoxicity tests were performed. During the experiment, extraction periods exceeding those prescribed by ISO 10993-5:2009 were employed to provide a more comprehensive assessment of the potential release of matrix and filler components. While some statistically significant differences were observed, cell viability remained above 70% across all experimental conditions and materials, indicating the absence of cytotoxic effects [44].

Mechanical properties are crucial for ensuring the safety of medical devices and fulfilling biomechanical design requirements. Among these properties, Shore A hardness is one of the most important parameters for polymeric materials used in mouthguards, occlusal splints, and aligners, as it is strongly correlated with the elastic modulus [89]. An increase in vinyl acetate content in EVA copolymers results in softer materials with a reduced elastic modulus [90], which allows for obtaining a range of materials that differ in this respect. For the applications under consideration, materials with hardness values ranging from 40 to 95 Shore A are employed [91], with values around 80 Shore A being the most common [92,93]; therefore, such material was selected as the reference for the experiments. It is important to note that hardness must be carefully adjusted and balanced: higher hardness values improve retention, shape stability, resistance to tribological wear, and the ability to generate appropriate orthodontic forces, while lower hardness values enhance patient comfort, facilitate intraoral adaptation, and reduce the risk of mucosal abrasions [3,91,94,95]. In the context of the present study, it is essential to maintain these original material properties.

The results showed a gradual increase in hardness, which additionally represents the growth of the elasticity modulus [89], with increasing SP concentration. Nevertheless, differences compared with the control material were small, with a maximum of 2.3 Shore A, and reached statistical significance only at 16% SP content. From the perspective of the intended applications, such subtle changes are fully acceptable and can be compensated for, for example, by blending with a copolymer containing a higher vinyl acetate content [96]. The observed trend is attributed to the restriction of polymer chain mobility caused by the presence of filler particles [97,98], which is consistent with reports for EVA-based composites [40] and other flexible polymers [72]. Importantly, the method of filler incorporation did not influence the hardness values.

The observed decrease in tensile strength at higher filler concentrations (EVA-C8 and EVA-C16) is consistent with previous reports on polymeric composites [99,100,101]. In parallel, for EVA-based composites, tensile strength generally remains unchanged, while toughness values—representing the energy absorbed before fracturing—increase for stiffer matrices but remain stable or even decrease for softer ones [102]. Our investigations have shown significant differences in toughness only between selected composites; the trends were similar to those observed for tensile strength. Nevertheless, standard deviations for toughness in the composites reached up to 13% of the average values, compared to 6% for tensile strength and 4% (for both properties) in neat EVA, which influenced the results of the statistical analyses. The potential mechanisms behind the observed changes have been widely discussed by Mariotti et al. [102]. They identify several ways in which fillers can enhance the mechanical strength of polymers, such as stress transfer from the matrix to the stiffer filler, improved energy absorption due to applied stress (allowing its dispersion across a larger volume of the composite matrix and thus increasing toughness), the formation of an interphase resulting from high interfacial areas and strong interactions between the matrix and the filler. Consequently, homogeneous dispersion and good adhesion between the matrix and the filler can lead to improvements in both tensile strength and toughness. However, in the case of softer matrices, these enhancements may be insufficient to improve the properties significantly. Conversely, weak interactions between the matrix and the filler promote the formation of micro-defects and delamination under load due to structural imperfections. Additionally, particle inhomogeneities, aggregation, or pore formation, often induced by increased viscosity of the filled system [103,104], can act as structural defects and stress concentrators, facilitating crack initiation and propagation under tensile loading.

For all composite materials, the values of the mechanical loss factor were similar to those of the EVA polymer, although they increased slightly at higher temperatures. At higher filler concentrations, both the storage modulus and the loss modulus increased. Similar simultaneous changes in G′ and G″ have been previously reported and are attributed to polymer–filler interactions, which restrict polymer chain mobility and increase energy dissipation due to friction within the rubber matrix [105,106]. Additionally, at low filler loadings, the softening effect may dominate over the weak reinforcing effect. When polymer–filler interactions are weak, the storage modulus may initially decrease [107], as observed in the current research. Weak interactions also result in stable tan(*δ*) values [107,108]. In the present study, the filler was not functionalized; hence, weak interactions were expected and considered acceptable, as the filler was introduced to improve microbiological rather than mechanical properties. An additional indication of weak interactions is the presence of microvoids formed around the filler particles [109], as observed in Figure 2f.

The contact angle measurements revealed values exceeding 90°, confirming the preservation of the hydrophobic nature of the base material. These results are consistent with those previously reported for EVA copolymers [110,111,112,113]. An increasing trend in contact angle values was observed with a higher filler content, which is advantageous, as enhanced hydrophobicity reduces bacterial adhesion [114,115,116].

Furthermore, incorporation of the filler into the matrix did not significantly affect the melting temperature, thereby ensuring that the material retained its original processability and thermoformability. The results of the analyses conducted thus far indicate that the melting temperature may change or remain unchanged, and the potential changes depend on the type and concentration of fillers. For nanocomposites comprising an EVA matrix and carbon black [117] or graphene oxide [118], a small reduction in Tm values was reported due to hindered chain mobility. Other studies have usually shown that loading fillers such as carbon nanofiller [119], barium titanate [102] microcrystalline cellulose [120] does not influence the melting temperature, which was attributed to a lack of changes in crystal type (even if overall crystallinity is changed, the existing crystals melt at the same temperature), unchanged lamellar thickness and the to the low filler–matrix interactions to change the overall melting behaviors.

A limitation of the present preliminary stage of the experiment is that only basic antibacterial and antifungal assays were conducted on freshly prepared samples. Future studies should verify whether the enhanced resistance to microorganisms is maintained in long-stored samples under humid environmental conditions, as this effect is expected to diminish over time due to the specific antimicrobial mechanism of the applied filler. Furthermore, future experiments should assess the material’s potential to prevent biofilm formation. This issue is further complicated by the fact that, under real-world conditions, surfaces are exposed to thermocycling, mechanical and chemical cleaning, as well as mechanical stresses—all of which can influence microbial survival. Additionally, these materials undergo tribological wear, which may expose underlying material layers and thereby “renew” the antimicrobial effect. However, the leaching of the filler from the matrix could reduce antimicrobial efficacy and alter the potential cytotoxicity of wear products. These aspects warrant further investigation.

## 5. Conclusions

Experimental composite materials modified with SP particles showed a significant reduction in colonies of pathogenic yeast-like fungi and cariogenic bacteria in the surrounding environment, without inducing cytotoxic effects and while preserving other analyzed biofunctional characteristics, including mechanical, physicochemical, and processing properties. However, further studies are required on material models under conditions that simulate long-term exposure to external factors to confirm the durability and stability of these properties over time.

## Figures and Tables

**Table 1:** The number of forming units of *S. mutans* ATCC 33535 and *C. albicans* ATCC 10231.

Material	Number of *S. mutans*, ×10^3^ CFU/mL				Number of *C. albicans*, ×10^3^ CFU/mL			
	Med.	IQR	Min.	Max.	Med.	IQR	Min.	Max.
EVA	23.8	2.2	26.05	16.35	12.45	2.9	9.15	16.65
EVA-M1	8.25	1.05	10	5.05	5.45	0.6	4.9	6.4
EVA-M16	4.6	0.2	6.05	4.4	0.15	0.35	0	0.45
EVA-C1	6.1	2.7	11.15	4.25	4.7	4.5	1.65	8.15
EVA-C2	5.55	0.7	6	4.7	4	3.25	2.75	8.1
EVA-C4	9.2	1.5	11.3	8.1	5.6	1.45	3.6	8.9
EVA-C8	10.95	1.4	12.45	8.55	3.6	2.05	1.1	4.85
EVA-C16	5.15	0.55	6.7	3.35	1.65	2.1	0.2	5.4

Med.—median value, IQR—interquartile range, Min.—minimum value, Max.—maximum value.

**Table 2:** Values of storage modulus (G′), loss modulus (G″), and mechanical loss factor (tgδ).

Material	23 °C			37 °C		
	G′, MPa	G″, MPa	tgδ	G′, MPa	G″, MPa	tgδ
EVA	7.23	0.52	0.07	4.50	0.41	0.09
EVA-M1	6.32	0.45	0.07	3.96	0.37	0.08
EVA-M16	8.59	0.59	0.07	5.35	0.45	0.08
EVA-C1	6.22	0.40	0.07	3.92	0.32	0.08
EVA-C2	6.35	0.43	0.07	4.00	0.34	0.09
EVA-C4	7.06	0.48	0.07	4.34	0.38	0.09
EVA-C8	7.72	0.54	0.07	4.95	0.42	0.09
EVA-C16	8.68	0.62	0.07	5.46	0.48	0.09

## Data Availability

The original contributions presented in this study are included in the article and Supplementary Materials. Further inquiries can be directed to the corresponding author.

## Supplementary Materials

The following supporting information can be downloaded at: https://www.mdpi.com/article/10.3390/ma18214993/s1. Figure S1: Comparison of the thermograms of (EVA) ant composites compounded with the solvent method (a) and mechanical mixing (b).

## Abbreviations

The following abbreviations are used in this manuscript:

SP	Silver sodium hydrogen phosphate, filler used in the experiment
EVA	Ethylene-vinyl acetate
G′	Storage modulus
G″	Loss modulus
tgδ	Loss factor
AME	Antimicrobial efficiency
CA	Water contact angle
TS	Tensile strength
CFU	Colony-forming units
DSC	Differential scanning calorimetry
