# Supplementary material for: Effect of Antimicrobial Filler on Ethylene-Vinyl Acetate (EVA) Composites Property
Source: Materials (Basel). 2025 Oct 31;18(21):4993. doi: 10.3390/ma18214993 (PMC12608532; doi:10.3390/ma18214993)
Supplement: Supplementary file 1 [file materials-18-04993-s001.zip › materials-3937907-supplementary.pdf]

Supplementary Materials

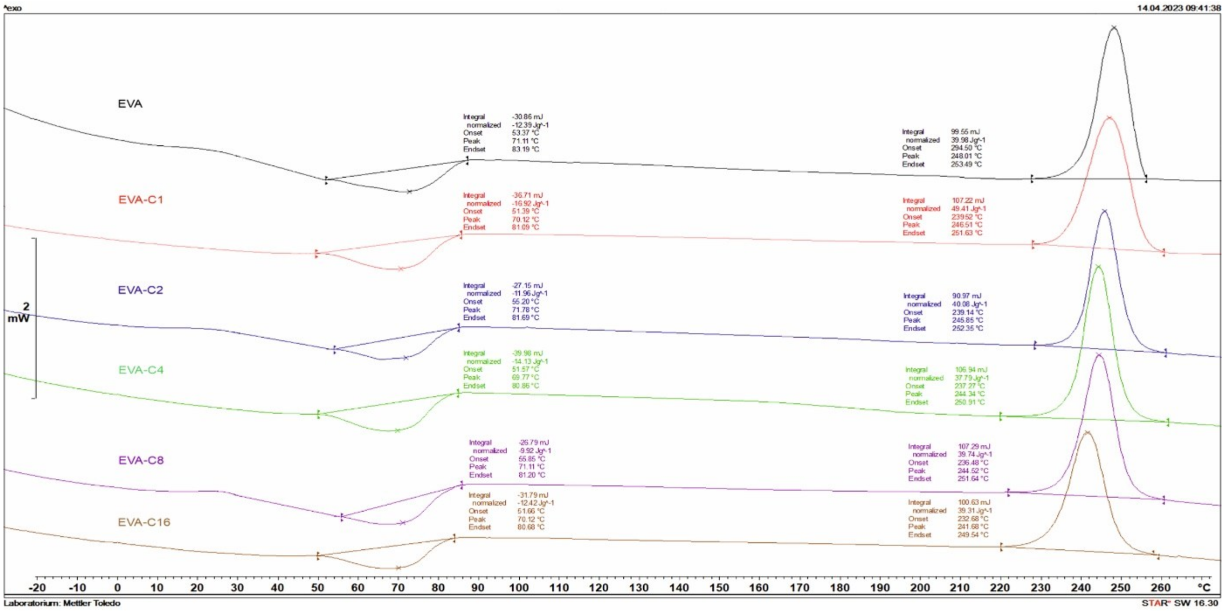

(**a**)

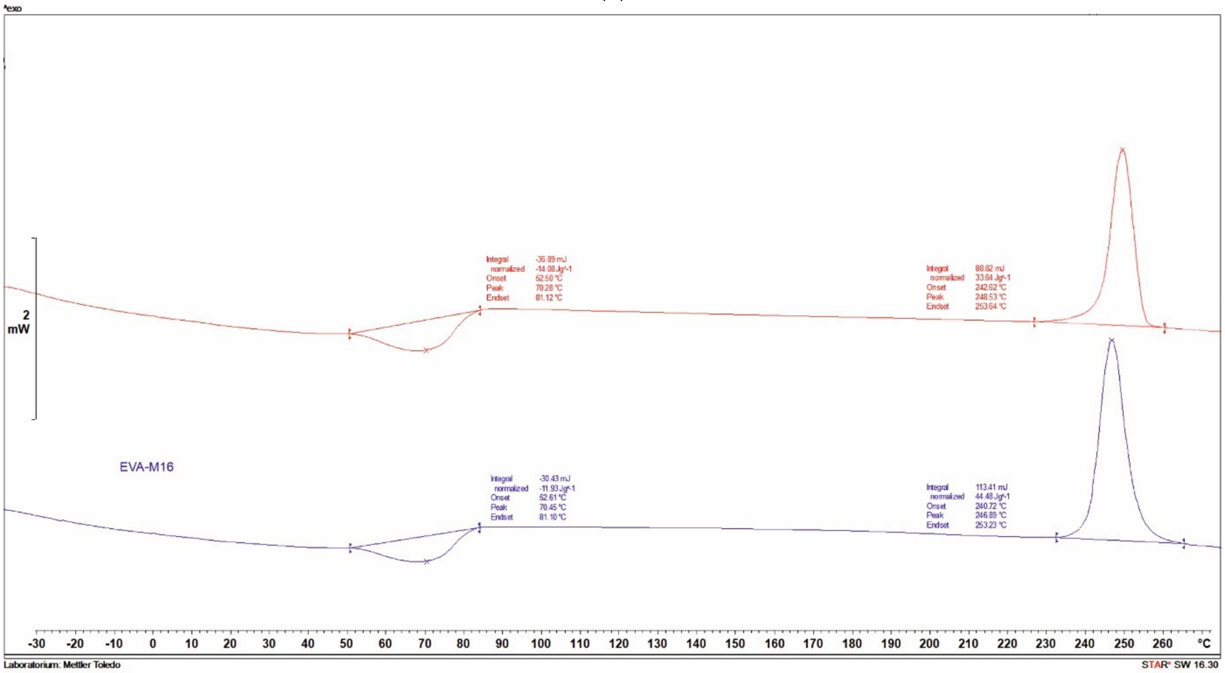

(**b**)

Figure S1. Comparison of the thermograms of (EVA) ant composites compounded with the solvent method **(a)** and mechanical mixing **(b)**.
